# Literary Notes

**Published:** 1901-05

**Authors:** 


### LITERARY NOTES.

Messrs. G. P. Engelhard & Co., Chicago, who are known as extensive medical publishers, announce the early publication of the Standard Medical Directory containing a complete list, revised to date, of the Physicians of the United States and Canada. Supplementing this important directory will be nine associate directories comprising a full list of all drugs and medicines in use; of hospitals and sanitariums; of medical publications and medical journals; medical officers of the army and navy, and of the marine hospital service; medical colleges and nurses' training schools; national, state, county and local medical societies; medical examining boards and medical laws; state boards of health; and a list of all manufacturers of products related to medicine.

The work will be issued in one volume of about 1,000 imperial octavo pages, printed on good quality of paper and bound in handsome library style. The work of compilation, in which the editors have the coöperation of the officers of the leading medical organisations of the country, is actively in progress and the directory is due to appear about July 15. Prompt responses by physicians to requests for individual data for entries are solicited by the management as a matter of mutual advantage. It is believed this great work will be a contribution of distinct value to the entire profession and its appearance will be awaited with interest.

---

THE Palisades Manufacturing Company, of Yonkers, has issued a description of the hermetic book of medicine of the ancient Egyptians, which was discovered by George Ebers during his visit to Egypt in 1872. Much of the hieroglyphic writing is reproduced upon an imitation of papyrus. It is an interesting publication and will be sent to any physician on application.

